# Protein Convertase Subtilisin/Kexin Type 9 Monoclonal Antibodies (PCSK9mab) in Clinical Practice at Secondary Care – Real World Multicentre Experience

**DOI:** 10.7759/cureus.33044

**Published:** 2022-12-28

**Authors:** Bilal Bashir, Shonagh Haslam, Shaheer Ahmad, Mohamed N Elnaggar, Rebecca Allcock, Sadaf Ali, Nyan M Kyi, Lorelei Salazar, Angela Gbegbaje, Moulinath Banerjee

**Affiliations:** 1 Endocrinology, Diabetes and Specialist Weight Management, Royal Bolton Hospital, Bolton, GBR; 2 Clinical Biochemistry, Lancashire Teaching Hospitals NHS Foundation Trust, Preston, GBR; 3 Endocrinology and Diabetes, University Hospitals of Morecambe Bay NHS Foundation Trust, Lancaster, GBR; 4 Endocrinology, Diabetes and Specialist Weight Management, Wrightington, Wigan and Leigh NHS Foundation Trust, Wigan, GBR; 5 Endocrinology and Diabetes, Lancashire Teaching Hospitals NHS Foundation Trust, Preston, GBR; 6 Chemical Pathology, Wrightington, Wigan and Leigh NHS Foundation Trust, Wigan, GBR; 7 Honorary Senior Lecturer, Edge Hill University Medical School, Ormskirk, GBR

**Keywords:** ldl-cholesterol, evolocumab, alirocumab, real world evidence, pcsk9 inhibitors

## Abstract

Background and Aims

Protein convertase subtilisin/Kexin type 9 monoclonal antibodies (PCSK9mab) are a novel addition to the therapeutic options for managing hyperlipidemia. Various guidelines have advocated the addition of these agents if the target low-density lipoprotein-cholesterol ( LDL-C) is not achieved by maximum lipid-lowering therapy. They have shown a robust and consistent reduction in LDL-C in clinical trials. However, the translation of these results in a real-world setting is limited and confined mainly to tertiary lipid centers. This service evaluation aimed to assess their efficacy in a real-world outpatient setting of secondary care centers.

Methods

Data was collected retrospectively from four hospitals in the North-West of England. Patients were required to attend a lipid clinic for follow-up investigations to continue with the prescription of PCSK9mab.

Results

A total of 175 patients were identified. Efficacy outcomes were measured in 169 patients. 6 discontinued the agent within 3 months of initiation and were excluded from the efficacy outcomes. 19.5% (n=33) had confirmed familial hypercholesterolemia. 61% (n=103) of the patients were intolerant to statins. 53.2% (n=90) of the patients have been prescribed Alirocumab. Mean LDL-C reduction was 50.6% at 6-month which was sustained at 48.9% at 12 months. There was no difference in % reduction of LDL-C between Alirocumab and Evolocumab. LDL-C reduction was more significant in patients who were on concomitant statins. 9.1% of patients experienced side effects, and 5.1% discontinued the PCSK9mab during treatment.

Conclusion

The efficacy of lipid reduction and the side effect profile of PCSK9mab from these secondary care services are similar to randomized clinical trials and real-world observational studies from tertiary lipid centers.

## Introduction

Hyperlipidemia has been established as a risk factor for atherosclerotic cardiovascular disease (ASCVD). There is unequivocal evidence that high levels of Low-Density Lipoprotein- Cholesterol (LDL-C) and other lipoproteins, including Very Low-Density Lipoprotein (VLDL), Intermediate Density Lipoprotein (IDL), and Lipoprotein (a) (Lp(a)) have an unfavorable effect on ASCVD and contribute to cardiovascular deaths [1]. The critical step in the genesis of ASCVD is the accumulation of cholesterol-rich lipoproteins in the arterial wall. Several epidemiological, genetic, mendelian randomized studies and randomized controlled trials (RCTs) evaluating the effect of lipid-lowering agents on ASCVD have provided consistent and unequivocal evidence of a causal relationship between hyperlipidemia and an increased risk of developing ASCVD and related mortality [1]and a linear and dose-dependent increase in the risk of ASCVD with the increase in total cholesterol [2]. The Cholesterol Treatment Trialists' (CTT) metanalysis of individual data from 27 randomized trials has demonstrated a 22% reduction in major vascular events with each 1 mmol/L reduction in LDL-C, even in those with no previous history of ASCVD [3].

Statins are regarded as the first line of pharmacotherapy, following lifestyle modifications for dyslipidaemias, and are considered the cornerstone in ASCVD prevention. On average, a high-intensity statin lowers the LDL-C by 50-60%, and a moderate-intensity by 30-50% [4]. Although there is interindividual variability of LDL-C reduction with the same statin dose, a poor response to statin treatment has been attributed partly to poor adherence and the genetic constitution of the patients [5]. Although statin-induced muscle symptoms exist in 15-20% of individuals, true statin intolerance leading to treatment discontinuation is seen in less than 5% of individuals [6]. However, in practice, the discontinuation rate of statins and non-adherence has been reported to be as high as 50% in the real-world setting [7-9]. Among the patients who cannot tolerate the recommended intensity of statin or who do not achieve the target reduction in cholesterol, the addition of a non-statin lipid-modifying agent is recommended [4].

PCSK9 monoclonal antibodies (PCSK9mab) are a novel addition to therapeutic options for patients with hypercholesterolemia. National Institute for Health and Care Excellence (NICE) recommends the use of PCSK9mab for primary and secondary prevention in primary familial hypercholesterolemia (FH), and for secondary prevention, in non-familial hypercholesterolemia (non-FH) and mixed dyslipidemia if they fail to attain target LDL-C with currently available therapies [10,11]. Two major cardiovascular outcome trials involving PCSK9mab have been conducted so far. Further Cardiovascular Outcomes Research with PCSK9 Inhibition in Subjects with Elevated Risk (FOURIER) trial with Evolocumab demonstrated a mean reduction of LDL-C of 59% at 48 weeks [12]. Evaluation of cardiovascular outcomes after an acute coronary syndrome during treatment with Alirocumab (ODYSSEY) trial demonstrated a mean reduction in LDL-C of 54.7% at 48 months [13]. Goal achievement after utilizing an anti PCSK9 antibody in statin-intolerant subjects-3 (GUASS-3) and ODYSSEY ALTERNATIVE studied the effect of Evolocumab and Alirocumab, respectively, in statin-intolerant subjects and demonstrated a 52.8% (49.8 - 55.8) and 45.0% (2.2) mean reduction in LDL-C, respectively [14,15]. Despite their promising results in these clinical trials, data on the performance of PSCK9mab in a real-world setting is limited and confined to tertiary lipid centers. Though RCTs demonstrate the efficacy of the drugs, a similar effect in the real world is not always seen. This could be due to patient selection, better adherence to the treatment plan, and adjunctive resources on health improvements that may be available to the trial participants [16]. Therefore, we assessed the efficacy and safety of PCSK9mab for a 12-month follow-up period at secondary care lipid centers and compared it to RCTs and currently available real-world data.

## Materials and methods

This was a retrospective multicentre service evaluation incorporating data from lipid clinics from four secondary care hospitals across the North-West of England. Between 2016-2020, patients were initiated on either of the available PCSK9mab (Evolocumab or Alirocumab) agents. The audit departments approved the project of the respective hospitals.

Inclusion criteria

Age>18 years, treatment with the locally available agent, either Evolocumab or Alirocumab, a continuation of the agent for at least 6 months, and be available for at least 1 follow-up set of investigations (after 6 or 12 months from initiation of treatment).

Exclusion criteria

Subjects with no follow-up results and subjects who discontinued the assigned PCSK9mab agent before the 6-month follow-up investigation were excluded from the analysis

Assessed lipid parameters included: Total Cholesterol (TC), Low-Density Lipoprotein (LDL), High-Density Lipoprotein (HDL), Triglycerides (TG), and non-HDL at baseline and 6 and 12 months after initiation of an agent were recorded. In addition, information regarding the patient's baseline macrovascular disease profile: (Ischemic Heart Disease (IHD), Cerebrovascular Accident ( CVA), Transient Ischemic Attack (TIA), Peripheral Vascular Disease (PVD), co-existence of other risk factors for vascular disease (Diabetes and hypertension), use of and tolerance to other concomitant lipid-lowering therapy were also recorded.

Primary outcome

Percent reduction in LDL-C as compared to baseline. The baseline was defined as LDL-C at the commencement of PCSK9mab therapy.

Secondary outcomes

Percent change in TC, non-HDL-C, TG, and HDL; The patient-reported side effects; Rate of discontinuation owing to side effects.

During the study period, PCSK9mab was initiated based on NICE guidelines [10-11] for FH, non-FH, or mixed dyslipidemia. The diagnosis of clinical FH was made based on Simon Broome's criteria or Dutch Lipid Clinic Network Score (DLCNS)[17]. Those meeting the criteria for probable/possible familial hypercholesterolemia were referred for genetic testing. Genetic testing facilities were unavailable at one center; therefore, the decision to treat FH clinically was based on Simon Broome's criteria alone. The decision to commence PCSK9mab in patients with mixed dyslipidemia with high triglycerides and incalculable LDL-C was based on total cholesterol of >7mmol/L for secondary prevention or using non-HDL cholesterol thresholds instead of LDL-C suggested by NICE guidance for secondary prevention or direct measurement of LDL-C.

Statistical analysis

The TC, LDL-C, HDL-C, non-HDL-C, and TG data were analyzed at baseline (before initiation of an agent), 6 and 12 months. Further subgroup analysis based on the type of agent used, statin tolerance, concomitant use of Ezetimibe in statin and statin-intolerant cohorts, and genetically confirmed FH was done. A one-way ANOVA was performed to compare baseline LDL-C, absolute LDL-C, and % LDL-C reduction between participating centers.

Continuous variables were summarized as mean (95% CI) for normally distributed data and median (Q1 - Q3) for skewed data. Categorical variables are reported as percentages or frequencies. Paired t-test was used to compare the absolute reduction and percent reduction at each follow-up time point of normally distributed data between baseline and follow-up time points within a subgroup. An unpaired t-test is used to compare change across different subgroups. For data with skewed distribution, Wilcoxon singed-rank test was used to compare baseline and follow-up time points within a subgroup, and the Kruskal Wallis test was used to analyze any differences across subgroups. Further observations were not recorded or analyzed if a PCSK9mab was discontinued at any time. Subjects with incalculable LDL-C due to high TG were not included in primary outcome analyses, and p <0.05 was considered statistically significant. Statistical analysis was performed using StatsDirect statistical software version 3.3.5.

## Results

Patient Characteristics

A total of 175 patients were identified; 6 discontinued medication before 3 months and were excluded from the efficacy analysis. Data of 169 patients had a mean age (95% CI) of 58.6 years (56.6 - 60.5), and 46.7% (n=79) were male. 60.9% (n=103) were intolerant to statins. The mean age (years) of patients with statin intolerance was higher than in patients who were statin-tolerant [61.4 (59.2 - 63.7) vs. 54.0 (50.7 - 57.2); p= <0.001]. Due to retrospective data analysis from different centers, we could not ascertain the precise criteria employed by individual centers to diagnose statin intolerance. Each center's lead prescriber confirmed that they had followed the NICE criteria in using these agents. The majority of subjects were prescribed PCSK9mab for secondary prevention [53.2% (n=90) vs. 46.7% (n=79)]. 42.6% (n=72) had a prior history of ischemic heart disease (IHD), 11.8% (n=20) had either a prior cerebrovascular accident (CVA) or transient ischemic attack (TIA) and 4.1% (n=7) had the peripheral vascular disease (PVD). Additional ASCVD risk factors, i.e., diabetes and hypertension, were present in 22.5% (n=38) and 35.5% (n=60), respectively. 53.3% (n=90) of the patients were on Alirocumab, while 46.7% (n= 79) were on Evolocumab (p=0.4). During treatment, four patients were switched from one PCSK9mab to the other. The cause for this was documented in only one case, and the patient is intolerant to the former. Genetic testing facilities were available in 3 out of 4 centers, identifying 33 patients with genetically confirmed FH. Amongst them, a majority had an LDL receptor mutation (93.9%, n=31), one involving the Apolipoprotein B gene (APOB) mutation and another with the gain-of-function mutation in the gene for PCSK9. All the subjects who were able to tolerate statins were on different statins at the beginning of the study, which included: Atorvastatin (53%, n =35), Rosuvastatin (38%, n=25), Simvastatin (3%, n=2) and unknown (6%, n=4). At baseline, 45.6% (n=77) of patients were on Ezetimibe; however, this was stopped in 13% (n=10) at the commencement of PCSK9mab. Ezetimibe was continued in 39.6% (n=67) of patients; 30 were statin intolerant (29.1% of the statin-intolerant cohort). The baseline cohort characteristics and treatment subgroups are summarized in Table 1.

**Table 1:** Demographics IHD: Ischemic Heart Disease; CVA/TIA: Cerebrovascular Accident/Transient Ischemic Attack; PVD: Peripheral Vascular Disease

Characteristics	Total (n=169)	Evolocumab (n=79)	Alirocumab (n=90)	Statin Tolerant (n=66)	Statin Intolerant (n=103)
Age (years)	58.6 (56.6 – 60.5)	58.0 (55.2 – 60.8)	59.1 (56.4 – 61.8)	54.0 (50.7 – 57.2)	61.4 (59.2 – 63.7)
Males (%)	79 (46.7)	37 (46.8)	42 (46.7)	31 (47.0)	48 (46.6)
Familial Hypercholesterolemia (%)	33 (19.5)	20 (25.3)	13 (14.4)	21 (31.8)	12 (11.6)
Primary Prevention (%)	79 (46.7)	36 (45.6)	43 (47.8)	37 (56.1)	42 (40.8)
Secondary Prevention (%)	90 (53.2)	43 (54.4)	47 (52.2)	29 (43.9)	61 (59.2)
Diabetes (%)	38 (22.5)	20 (25.3)	18 (20)	9(13.6)	29 (28.1)
Hypertension (%)	60 (35.5)	29 (36.7)	31 (34.4)	19 (28.8)	41 (39.8)
IHD (%)	72 (42.6)	36 (45.6)	36 (40)	25 (37.9)	47 (45.6)
CVA/TIA (%)	20 (11.8)	10(12.6)	10 (11.1)	7 (10.6)	13 (12.6)
PVD (%)	7 (4.1)	6 (7.6)	1 (1.1)	1 (1.5)	6 (5.8)
Statins (%)	66 (39)	30 (38)	36 (40)	66 (100)	-
Ezetimibe (%)	67 (39.6)	27 (34.2)	40 (44.4)	37 (56.1)	30 (29.1)

Efficacy outcomes

**Figure 1:**
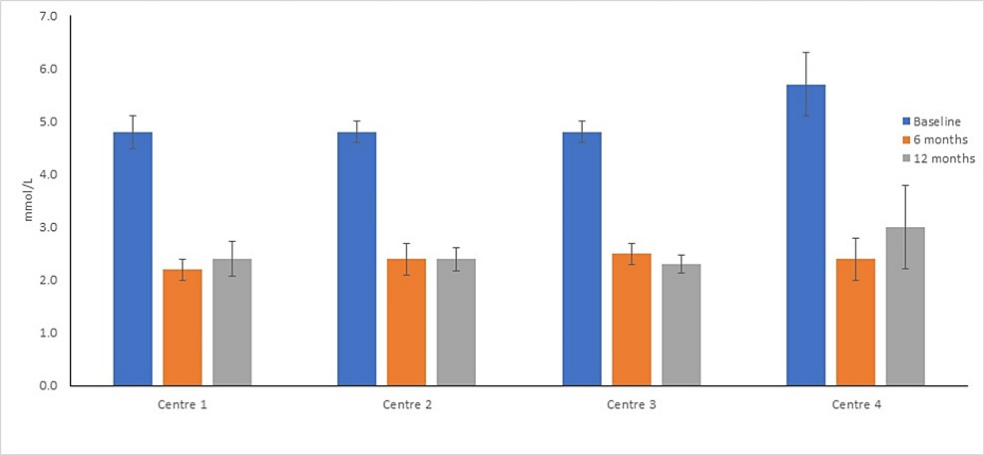
Comparison of baseline, 6 months, and 12 months follow-up LDL-C between centers. No statistically significant difference was observed in baseline, 6 months, 12 months, absolute reduction, and % reduction of LDL-C between individual centers. LDL-C: low-density lipoprotein- cholesterol.

There was no statistically significant difference in baseline (F=0.72, p=0.5), 6 months (F=0.24, p=0.87), and 12 months (F=0.47, p= 0.7) of LDL-C between centers. Absolute and % reduction at 6 months (F= 1.6 and 1.1, p= 0.2 and 0.34) and 12 months (F=1.1 and 0.16, p= 0.36 and 0.92) between centers also showed no differences from each other (one way ANOVA) (Figure 1).

Effect on LDL-C

**Figure 2:**
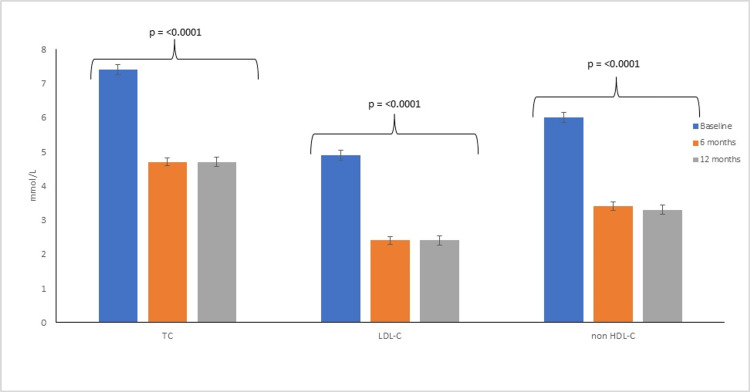
Efficacy of PCSK9i on Total Cholesterol (TC), Low-density lipoprotein (LDL-C), and non- High-density lipoprotein (non-HDL-C) TC: Total Cholesterol; LDL-C Low-density lipoprotein- cholesterol; non-HDL-C: High-density lipoprotein- cholesterol

The mean (95% CI) LDL-C (mmol/L) at baseline for the study population was 4.9 (4.6 - 5.2), which was reduced to 2.4 (2.2 - 2.6), which was sustained at 2.4 (2.1 - 2.6) at 12 months [% reduction of 50.6% (46.5 - 53.8) and 48.9% (44.3 - 53.4), respectively] (Figure 2, Table 2). During treatment, there were a significant number of subjects who achieved LDL-C reduction of >50% from baseline at 6 months (55.9%, n = 76) and 12 months (51.4%, n= 56), and >40% from baseline at 6 months (68.4%, n=93) and 12 months (66.1%, n=72). 37.5% (n=51) and 41.2% (n=45) subjects achieved LDL-C <1.8 mmol/L, 23.5% ( n=32) and 22.9% (n=25) achieved LDL-C <1.4 mmol/L at 6 and 12 months, respectively. The percent reduction in LDL-C with Evolocumab and Alirocumab was similar at 6 months [51.2 (45.3 - 57.1) and 49.3 (44.5 - 54.0) respectively, p=0.6) and 12 months [52.7 (44.3 - 59.8) and 47.1 (41.3 - 52.8), respectively, p = 0.3) (Table 5 in Appendices). The use of adjunctive lipid-lowering medication, i.e., statins at baseline with monoclonal antibodies, has been associated with a greater reduction in mean LDL-C at 6 months (55.2% (49.4 - 61.0) vs. 47.5% (42.9 - 52.3), p= 0.05) which was maintained at 12 months (53.6% (45.4 - 61.9) vs. 46.1% (40.7 - 51.5), p= 0.1) (Table 6 in Appendices). In subjects taking and continuing statins from baseline, addition/continuation of Ezetimibe did not demonstrate any additional percentage reduction in LDL-C at 6 months [57.9% (50.5 - 65.2) and 52.6% (38.7 - 66.1), p= 0.4] or 12 months [49.7% (39.0 - 60.3) and 61.0 (47.4 - 74.6), p= 0.2]. Although the trend of LDL-C reduction with concomitant use of Ezetimibe was reversed at 12 months, it did not reach the level of significance; however, the sample size for this cohort was small (n= 26 for 6 months and n=14 for 12 months) (Table 7 in Appendices). In the statin-intolerant cohort, absolute and % reduction of LDL-C was numerically but not significantly higher in patients who did not have concomitant exposure to Ezetimibe at 6 months; however, it was similar at 12 months ( p= 0.1) (Table 8 in Appendices)

**Table 2:** Efficacy outcomes (Whole cohort) a: 6-month cohort after excluding subjects with TG > 4.5 mmol/L b: 12-month cohort after excluding subjects with TG > 4.5 mmol/L c:  p= < 0.0001 d: p= <0.005 TC: Total Cholesterol; LDL-C: Low-density lipoprotein- cholesterol; HDL-C: High-density lipoprotein-cholesterol; TG- Triglycerides

Variable (mmol/L)	Baseline	6 months (n= 155)	12 months (n= 126)	Absolute reduction (6 months)	Absolute reduction (12 months)	% Reduction (6 months)	% Reduction (12 months)
TC	7.4 (7.1 – 7.7)	4.7 (4.5 – 5.0)	4.7 (4.4 – 4.9)	2.6 (2.4 – 2.8)	2.5 (2.2 – 2.8)	34.6 (31.9 – 37.2)^ c^	34.1 (30.6 – 37.7)^ c^
LDL-C (n=136^a^, n=109^b^)	4.9 (4.6 – 5.2)	2.4 (2.2 – 2.6)	2.4 (2.1 – 2.6)	2.4 (2.2 – 2.7)	2.4 (2.1 – 2.7)	50.6 (46.5 – 53.8)^ c^	48.9 (44.3 – 53.4)^ c^
HDL-C	1.4 (1.3 – 1.5)	1.4 (1.3 – 1.5)	1.3 (1.2 – 1.4)	-	-	-	-
Non-HDL-C)	6.0 (5.7 – 6.3)	3.4 (3.1 – 3.6)	3.3 (3.0 – 3.6)	2.6 (2.4 – 2.8)	2.5 (2.3 – 2.8)	42.9 (39.6 – 46.2)^ c^	42.0 (37.8 – 46.3)^ c^
TG	2.0 (1.3 – 3.4)	1.7 (1.2 – 2.9)	1.8 (1.2 – 2.7)	0.2 (-0.2 – 0.8)	0.3 (-0.2 – 0.9)	12.2 (-12.5 – 35)^ d^	15.7 (-15.7 – 36.9)^ c^

Effect on LDL-C levels in Confirmed Familial Hypercholesterolemia by genetic analysis

There were 33 subjects with genetically proven FH in this cohort. Six-month follow-up results were available for 29 subjects, and 12-month follow-up data for 23 subjects (Table 3).

**Table 3:** Efficacy outcomes in Familial Hypercholesterolemia a: p= < 0.0001 b: p= < 0.005 c: NS TC: Total Cholesterol; LDL-C: Low-density lipoprotein- cholesterol; HDL: High-density lipoprotein-cholesterol; TG: Triglycerides

Variable (mmol/L)	Baseline	6 months (n= 29)	12 months (n= 23)	Absolute reduction (6 months)	Absolute reduction (12 months)	% Reduction (6 months)	% Reduction (12 months)
TC	7.4 (6.5 – 8.2)	4.3 (3.7 – 4.9)	4.5 (3.8 – 5.2)	3.0 (2.4 – 3.6)	2.6 (1.9 – 3.4)	40.6 (34.8 – 46.3)^a^	35.5 (26.4 – 44.6)^a^
LDL-C	5.3 (4.6 – 6.1)	2.5 (1.9 – 3.1)	2.6 (1.9 – 3.2)	2.8 (2.3 – 3.3)	2.5 (1.8 – 3.2)	54.5 (47.7 – 61.4)^a^	48.1 (36.9 – 59.4)^a^
HDL-C	1.3 (1.2 – 1.4)	1.3 (1.2 – 1.4)	1.3 (1.2 – 1.4)	-	-	-	-
Non-HDL-C	6.1 (5.2 – 6.9)	3.0 (2.4 – 3.6)	3.2 (2.5 – 3.8)	3.0 (2.5 – 3.6)	2.7 (2.0 – 3.4)	50.8 (44.3 – 57.2)^a^	44.2 (33.7 – 54.6)^a^
TG	1.4 (0.9 – 2.1)	0.9 (0.8 – 1.4)	1.1 (0.9 – 1.8)	0.3 (0.1 – 0.7)	0.2 (-0.1 – 0.7)	25.1 (5.4 – 45.4)^b^	20.0 (-15.7 – 33.9)^c^

The mean (95% CI) LDL-C (mmol/L) at baseline was 5.3 (4.6 - 6.1), which reduced to 2.5 (1.9 - 3.1) at 6 months, with a mean reduction of 54.5% (47.7 - 61.4), p = <0.0001. 58.6 % (n=17) of patients achieved LDL-C reduction of >50% and 44.8% (n=13) subjects achieved LDL-C levels of <1.8mmol/L. 55.2% (n = 16) of subjects from the FH cohort received Evolocumab. The mean (95% CI) reduction in LDL-C (mmol/L) in subjects receiving Evolocumab was 54.6% (43.9 - 65.2), which was comparable to a mean (95% CI) LDL-C (mmol/L) reduction in LDL-C achieved by Alirocumab, 54.5% (44.8 - 64.2), p= 0.99. Similar trends of LDL-C reduction were observed based on statin intolerance, where subjects treated with concomitant statins (n=18) exhibited a greater mean percent reduction in LDL-C compared to subjects who were intolerant to statin (n = 11). However, this comparison did not reach the level of significance [59.1 (50.0 - 68.1) vs. 47.2 (36.5 - 57.8), p=0.08]. The mean (95% CI) LDL-C (mmol/L) at 12 months was 2.6 (1.9 - 3.2), which was comparable to the mean LDL-C at 6-month follow-up, p = 0.8. In contrast to the 6-month follow-up results, the mean (95% CI) % reduction in LDL-C in subjects receiving Evolocumab (n=14) was numerically greater than the subjects receiving Alirocumab (n=9); however, it did not reach the level of statistical significance [55.8 (42.1 - 69.4) vs. 36.3 (15.9 - 56.6), p= 0.13]. Similarly, the degree of % LDL-C reduction was similar between the statin tolerant (n=14) [53.4 (35.6 - 71.1) and intolerant patients (n=9) [40.0 (28.8 - 51.2) of FH cohort (p= 0.17).

Effect on Total Cholesterol

The effect of PCSK9mab on total cholesterol mirrored the observations of LDL-C. The mean (95% CI) TC (mmol/L) at baseline was 7.4 (7.1 - 7.7), which was reduced to 4.7 (4.5 - 5.0) at 6 months and was sustained at 4.7 (4.4 - 4.9) at 12 months. This corresponded to a mean % reduction of 34.6 (31.9- 37.1) and 34.1 (30.6 - 37.7) at 6 and 12 months, respectively (p = <0.0001) (Table 2). Both PCKS9 monoclonal antibodies, Evolocumab and Alirocumab, showed comparable mean % reduction at 6 months [34.8 (30.4 - 39.2) vs. 34.3 (31.0 - 37.4), p= 0.84] and 12 months [34.3 (28.1 - 40.6) vs. 34.0 (29.6 - 38.4), p= 0.93] ( table 5 in Appendices). Concomitant statin treatment with PCSK9mab led to numerically but not significantly a more significant reduction in TC at 6 and 12 months of follow-up compared to the statin-intolerant cohort. (p= 0.16 and 0.12, respectively) (Table 6, 7)

Effect on non-HDL cholesterol

The efficacy outcomes of PCSK9mab on non-HDL cholesterol were similar to the observed efficacy in reducing TC and LDL-C. We observed a mean reduction of 42.9% (39.6 - 46.2) in non-HDL-C at 6 months of follow-up that was sustained at 42.0% (37.8 - 46.3) at 12 months (Table 2). The effect of PCSK9mab on non-HDL in subgroup analysis mirrored the efficacy outcomes of LDL-C and TC (Table 5-8 in Appendices).

**Figure 3:**
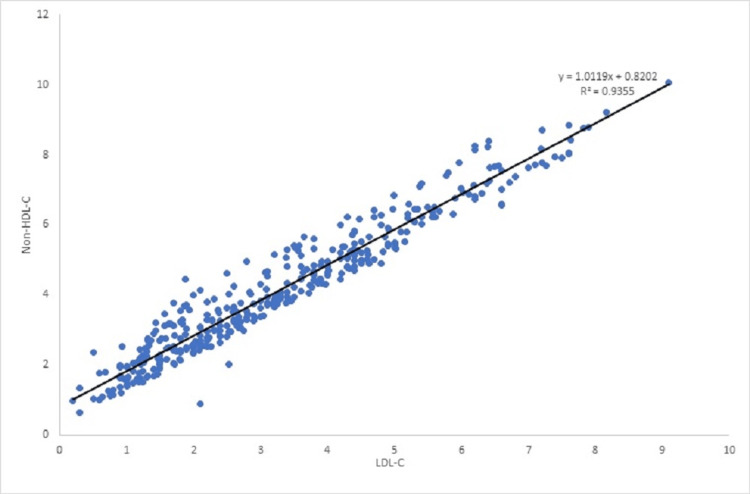
Correlation between LDL-C and Non-HDL-C at baseline, 6 months, and 12 months LDL-C: Low-density lipoprotein- cholesterol; non-HDL-C: High-density lipoprotein-cholesterol

Non-HDL-C was used as a surrogate marker for LDL-C cholesterol for diagnostic and monitoring purposes at one center if LDL-C is incalculable due to hypertriglyceridemia. We found a good correlation between non-HDL cholesterol and LDL-C at baseline, 6 months, and 12 months (r=0.97, p-<0.0001) (Figure 3); however, we are unable to establish if these findings can be extrapolated to subjects with high triglyceride levels (>4.5 mmol/L).

Effect on HDL-C and Triglycerides

Triglyceride levels (mmol/L) reduced from a median (Q1-Q3) of 2.0 (1.3 - 3.4) to 1.7 (1.2 - 2.9) at 6 months which was sustained at 1.8 (1.2 - 2.7) at 12 months, corresponding to a median% reduction of 12.5% (-12.8 - 35.0), p= 0.0002 at 6 months and 15.7 (-15.7 - 36.9), p= <0.0001 at 12 months. 12.3% (n=19) of the patients had a baseline TG >4.5 mmol/L. Reduction in TG was comparable in both PCSK9mab groups at 6 months (p= 0.88) and 12 months (p= 0.15). Similarly, no significant difference was observed between subgroups using adjuvant lipid-lowering therapy, i.e., statins or intolerant to statins at 6 months (p= 0.46) or 12 months (p=0.08) (Table 5-8 in appendices).

In contrast to other variables, HDL-C did not show any change with PCSK9mab usage but sustained its level in the first 12 months of treatment (Table 2, 3, 5-8).

Safety Profile

In 4 patients, the PCSK9mab was altered during the follow-up period. We could not ascertain the reason for drug alteration in 3 patients who were switched from Alirocumab to Evolocumab; however, no side effects were documented in these patients. In one patient, Evolocumab was changed to Alirocumab after the patient reported gingivitis attributed to Evolocumab. 5.1% (n=9) patients discontinued PCSK9mab, 6 within 6 months of initiation and remaining (n=3) between 6 to 12 months. Efficacy outcomes were not measured after the discontinuation of the agent. The spectrum of side effects that led to the discontinuation of the agent was arthralgias, myalgias, migraine, rash, vomiting, dizziness, mood disturbance, and hepatotoxicity. Side effects related to arthralgias and myalgias were mild in 3 patients, which did not lead to discontinuation of the medication. One patient experienced dose-dependent peripheral neuropathy; and continued a lower dose of Alirocumab. Almost all the patients (81.2%, n=13) who experienced side effects with PCSK9mab were intolerant to statins. Side effects associated with PCSK9mab and the reason for discontinuation have been summarized in Table 4.

**Table 4:** Safety Profile

Side effect	Number	Agent Discontinued
Arthralgias & Myalgias	6	3
Rash	2	1
Low mood, Insomnia	1	1
Hepatotoxicity	1	1
Memory deficit	1	1
Peripheral Neuropathy	1	No – dose-dependant
Gingivitis	1	0
AE not specified	1	1
Nausea & Diarrhoea	1	1
Temporal Headache, polyuria	1	0
Total	16	09

## Discussion

This real-world analysis evaluated the efficacy and safety outcomes of PCSK9mab from four hospitals. This is the first-ever and most extensive study evaluating the long-term real-world outcomes of PCSK9mab outside of the tertiary care settings in the UK. In our study, most patients were intolerant to statins (61%), which appears to be the main driver of the initiation for PCSK9mab. They were prescribed almost equally for primary prevention (46.7%) and secondary prevention (53.2%). We found a 50.6% reduction in LDL-C after the initiation of PCSK9mab, where either agent, Alirocumab or Evolocumab, showed comparable LDL-C reduction (51.2% and 49.3%). We found a more significant reduction in LDL-C in subjects who were not intolerant to statins and had concomitant statin treatment (55.2%) compared to the statin-intolerant cohort (47.5%). Continuation of another lipid-lowering adjunctive (Ezetimibe) was found to have additional beneficial effects at the 6-month follow-up; however, this trend was seen to be reversed at 12 months. A plausible explanation for this could be a higher baseline LDL-C level in the cohort who was not the recipient of Ezetimibe; however, the sample size for this cohort was too small to draw a definite conclusion.

Two large RCTs have evaluated the efficacy of the two commercially available PCSK9mab. In these trials, further cardiovascular outcome research with PCSK9 inhibition in subjects with elevated risk (FOURIER) [12] has demonstrated a mean LDL-C reduction of 59% (58 - 60) at 12 months, and ODYSSEY outcomes [13] have demonstrated a mean LDL-C reduction of 61% at 12 months with 54.7% at 48 months. The authors demonstrated that 87% of the subjects achieved an LDL-C reduction of <1.8 mmol/L. Our study demonstrated results consistent with both significant RCTs regarding the mean percent reduction of LDL-C; however, the proportion of patients achieving LDL-C < 1.8 mmol/L was lower than FOURIER and ODYSSEY. In contrast to most clinical trial settings, where the patients were treated with more intensive lipid-lowering therapy (LLT), our patients were treated to achieve either NICE or ESC targets. The majority of study subjects in the FOURIER trial and ODYSSEY OUTCOMES trial were on high-intensity (69.5% and 88.6%, respectively) or moderate or low-intensity statins (30.5 and 8.8%, respectively) along with PCSK9mab with or without Ezetimibe; whereas, in our study population only 39% of subjects had concomitant statin treatment. As a part of local practice, one center stopped Ezetimibe at the commencement of PCSK9mab, preventing the total intensification of LLT.

Furthermore, the lack of definitive criteria to define statin intolerance during the time frame in which this cohort was managed and inconsistencies in the provision of statin re-challenge with the different agents were the main drivers behind the more significant proportion of patients who were not on concomitant statin therapy. In contrast to our study cohort, baseline mean LDL-C in these RCTs was about 50% lower than in our study population, explaining a more significant proportion of patients achieving LDL-C <1.8 mmol/L. Intense monitoring, appropriate, monitored injection technique, a more significant proportion of concomitant statin therapy, standardized lipid-modifying diet, and difference in baseline characteristics of the study population might explain the differences in LDL-C reduction in RCT study subjects against our real-world cohort. In addition to LDL-C, non-HDL-C and TC also showed comparable reductions with PCSK9mab compared to the FOURIER trial, which has demonstrated a mean reduction of 51.2% in non-HDL-C as compared to 42.5% in our cohort, and a 35.5% reduction in TC as compared to 34.3%. Our cohort for subgroup analysis of Evolocumab did not show any significant change in TG or HDL-C, whereas, Evolocumab recipients in the FOURIER trial demonstrated a significant increase in HDL-C and reduction in TG.

61% of our study cohort were statin intolerant and demonstrated a mean LDL-C reduction of 47.5% (42.9 - 52.3) at 6 months that was sustained at 46.1% (40.7 - 51.5) at 12 months. Moriarty et al. [15] demonstrated a 45.0% (2.2%) reduction in mean LDL-C achieved with Alirocumab in a statin-intolerant cohort of 126 subjects assessed at 24 weeks. Similarly, Nissen et al. [14] have demonstrated a 52.8% (49.8 - 55.8) reduction in LDL-C with Evolocumab in a statin-intolerant cohort of 145 statin-intolerant subjects, measured at 24 weeks. Both of these study cohorts recruited patients for primary and secondary prevention and had baseline LDL-C comparable with our study cohort [4.9 (1.9) mmol/L and 5.6 (1.9) mmol/L, respectively]. Efficacy outcomes for other lipid parameters, i.e., non-HDL-C (mmol/L) [40.2 (1.7) and 45.7 (43.1 - 48.3)] and TC (mmol/L) [31.8 (1.4) and 36.6 (34.5 - 38.8)] were also comparable with our cohort. In contrast to the FOURIER and ODYSSEY trials, GUASS3 and ODYSSEY ALTERNATIVE did not show a significant reduction in TG, and ODYSSEY ALTERNATIVE did not show a significant change in HDL-C, consistent with our results.

The most extensive real-world data with PCSK9mab were reported from the Netherlands by Stoekenbroek et al., that evaluated data from 238 patients [18]. They reported a mean LDL-C reduction of 55.0% (52.3-57.7), comparable to our cohort, where the mean reduction in LDL-C was 50.6% (46.5 - 53.8). Baseline LDL-C was slightly higher in our cohort [4.4 (3.4-5.7) vs. 4.9 (4.6 - 5.2)] [18]. Efficacy outcomes in other lipid parameters, i.e., TC (mmol/L) and non-HDL-C (mmol/L), were also comparable with our data [35.4 (33.1-37.7) vs. 34.6 (31.9 - 37.2) and 48.1 (45.3-51.0) vs. 42.9 (39.6 - 46.2), respectively]. Our study did not demonstrate a significant rise in HDL-C but showed a 12.2% reduction in TG compared to the 7% reduction demonstrated by Stockenbroel et al. The trend of achieving a higher mean % reduction in LDL-C with concomitant statin treatment has previously been reported and replicated by Boers et al. (47% vs. 58%, P = 0.03) [19], Rallidis et al. (57.2% vs. 51.8%, p = 0.09) [20]and Hollstein et al. (60.2% vs. 53.1%, p <0.05) [21]. The efficacy in lowering LDL-C was similar amongst patients with and without FH, as reported in previous studies [20,22].

We demonstrated guideline-recommended LDL-C reduction [4] by 50% from baseline in 55.9% and LDL-C <1.8 mmol/L in 37.5% of the patients sustained at a 50% reduction in 51.4% and mean LDL-C <1.8 mmol/L in 41.2% of the patients. This was lower than the cohort that achieved the target LDL-C of <1.8 mmol/l, as reported by studies from the Netherlands [18] and Greece [20]. However, this was higher than the reported percentage of subjects achieving mean LDL-C <2 mmol/L (20%) in a cohort of 105 patients from the UK [23]. Similarly, another study from the UK by Han et al. [24] showed that 27.8% of the cohort achieved an LDL-C of <1.8 mmol/L, and 56.6% of the subjects achieved an LDL-C reduction of >50%, consistent with our results. The greater proportion of patients achieving a target LDL-C of <1.8 mmol/L from other countries could be explained by a greater proportion of study subjects on adjunctive lipid-lowering therapy, i.e., statins and Ezetimibe, which was a pre-requisite to acquiring PCSK9mab prescription in the Netherlands and Greece, which encompassed a lower proportion of statin-intolerant individuals and lower baseline LDL-C as compared to our cohort. Similarly, the proportion of patients who failed to achieve LDL-C reduction of <30% in our cohort (14.7%) was slightly higher as compared with other real-world retrospective data from Europe (11.3% and 10.6%) [20,22] but was comparable with real-world observational studies from the UK (13%), where hypo-responders are defined as LDL-C reduction <25% [23].

In our clinical setting, the discontinuation rate of PCSK9mab was 5.3% which was lower than other similar studies from the UK (15 & 17.6%)[23,25] but comparable to other European real-world studies, where the discontinuation rate ranges from 2.5% - 10% [18-20]. 9.1% of the patients reported side effects, and the spectrum of side effects was comparable to other real-world studies [18,20,22,23].

To date, the total body of evidence to demonstrate the efficacy of PCSK9mab in routine care is sparse. The most extensive retrospective analysis from the UK was a two-center study comprising a tertiary and a secondary care center, with results comparable to ours. Similarly, the largest European study originated from a tertiary referral center in Amsterdam. For the first time, we have demonstrated the use and efficacy of PCSK9mab outside the tertiary lipid centers in secondary care centers and achieved similar results. LDL-C reduction is crucial in reducing cardiovascular mortality and morbidity for FH and non-FH patients with ASCVD. Chamberlain et al., after reviewing the dataset of >3.5 million individuals, demonstrated low overall usage of PCSK9mab due to a lack of outcome data, prior-authorization approval, and cost implications [26]. Similar findings were reported by Knowles et al. [27], in which concerns were raised regarding a higher rejection rate for PCSK9mab prescriptions. Our report highlights and emphasizes the safety and efficacy of PCSK9mab and suggests that its use should not be restricted to tertiary lipid centers but should also be considered in secondary care centers, where adequate expertise for initiating and monitoring such specialist treatment is available.

Limitations

Despite being the largest real-world retrospective analysis of PCSK9mab usage from the UK, where participation from four different centers has provided data from a heterogeneous population that may have a close reflection of the region's population, there are inherent limitations to our study. These were mainly due to local variations in practice. One center stopped Ezetimibe on the commencement of PCSK9mab, thereby preventing the patients from experiencing maximum lipid-lowering benefits. Genetic testing was unavailable at one center; therefore, patients were treated based on a clinical diagnosis of FH according to Simon Broome's criteria. Details about statin intolerance were not available. At the time of the study, there were no uniform accepted criteria to define statin intolerance amongst all centers. Therefore, information on intolerance to statins or re-challenge with a different statin was unavailable. Since then, NICE has qualified the definition in their TA694. Many patients had mixed dyslipidemia with TG >4.5 mmol/L leading to incalculable LDL-C, and facilities to measure direct LDL-C were unavailable. There was no uniform policy to decide on the commencement of PCSK9mab in this subset of patients. A substantial number of patients did not have follow-up blood investigations at 12 months due to the disruption of services because of the COVID-19 pandemic. All the data were collected during routine clinical care in a retrospective fashion; therefore, adherence, medication compliance, and injection techniques could not be evaluated, and ASCVD risk stratification (very high, High, Moderate, Low) was not possible in all cases within this study.

## Conclusions

Our real-world data has demonstrated efficacy and a side effect profile with PCSK9mab comparable to large clinical trials and a limited number of available real-world studies. For the first time, we have demonstrated similar efficacy and a safety profile with these agents outside the tertiary care centers using the largest patient database of FH and non-FH patients in the UK. Therefore, we emphasize the need for the health care system to facilitate drug prescription, approval, and continuation in secondary and primary care.

## Appendices

**Table 5:** Efficacy outcomes of Evolocumab and alirocumab a: 6-month cohort after excluding subjects with TG > 4.5 mmol/L b: 12-month cohort after excluding subjects with TG > 4.5 mmol/L c:  p= < 0.0001 d: p= <0.005 e: NS TC: Total Cholesterol; LDL-C: Low-density lipoprotein- cholesterol; HDL-C: High-density lipoprotein-Cholesterol; TG: Triglycerides; NS: Not Significant

Variable (mmol/L)	Baseline	6 months	12 months	Absolute reduction (6 months)	Absolute reduction (12 months)	% Reduction (6 months)	% Reduction (12 months)
Evolocumab (6months n= 70, 12 months n= 49)							
TC	7.4 (7.0 – 8.0)	4.8 (4.4 – 5.1)	4.7 (4.2 – 5.2)	2.7 (2.3 – 3.1)	2.7 (2.1 – 3.2)	34.8 (30.4 – 39.2)^ c^	34.3 (28.1 – 40.6)^ c^
LDL-C (n=62^ a^, n=40^b^)	5.0 (4.5 – 5.5)	2.4 (2.0 – 2.8)	2.4 (1.9 – 2.9)	2.5 (2.2 – 2.9)	2.6 (2.1 – 3.1)	51.2 (45.3 – 57.1)^ c^	52.7 (44.3 – 59.8)^ c^
HDL-C	1.4 (1.3 – 1.5)	1.4 (1.3 – 1.6)	1.3 (1.2 – 1.4)	-		-	-
nHDL-C	6.1 (5.6 – 6.6)	3.3 (2.9 – 3.7)	3.3 (2.9 – 3.8)	2.8 (2.4 – 3.2)	2.7 (2.2 – 3.2)	44.2 (38.5 – 50.0)^ c^	42.5 (35.0 – 50.1)^ c^
TG	2.1 (1.3 – 3.6)	1.8 (1.1 – 2.7)	1.9 (1.2 – 2.8)	0.3 (-0.2 – 0.9)	0.2 (-0.5 – 0.8)	17.3 (-10.5 – 42.5)^ d^	13.3 (-28.6 – 32.4)^ e^
Alirocumab (6months n= 85, 12 months n=77)							
TC	7.3 (6.9 – 7.7)	4.7 (4.4 – 5.1)	4.6 (4.2 – 5.0)	2.6 (2.2 – 2.8)	2.5 (2.1 – 2.8)	34.3 (31.0 – 37.4)^ c^	34.0 (29.6 – 38.4)^ c^
LDL-C (n=74^a^, n=69^b^)	4.7 (4.4 – 5.1)	2.4 (2.1 - 2.7)	2.4 (2.1 -2.7)	2.3 (2.0 – 2.6)	2.3 (1.9 0 2.6)	49.3 (44.5 – 54.0)^ c^	47.1 (41.3 – 52.8)^ c^
HDL-C	1.4 (1.3 – 1.5)	1.3 (1.2 – 1.4)	1.3 (1.2 – 1.4)	0.06 (0.02 – 0.1)	-	4.0 (0.9 – 7.1)^ d^	-
nHDL-C	5.9 (5.5 – 6.2)	3.4 (3.1 – 3.7)	3.3 (2.9 – 3.6)	2.5 (2.2 – 2.8)	2.4 (2.1 – 2.8)	41.7 (37.9 – 45.6)^ c^	41.7 (36.6 – 46.8)^ c^
TG	2.0 (1.3 – 3.4)	1.6 (1.2 – 2.9)	1.8 (1.2 – 2.5)	0.2 (-0.2 – 0.7)	0.3 (-0.1 – 1)	11.1 (-13.2 – 34.1)^ d^	20.0 (-9.1 – 37.5)^ c^

**Table 6:** Efficacy outcomes based on statin tolerance a: 6-month cohort after excluding subjects with TG > 4.5 mmol/L b: 12-month cohort after excluding subjects with TG > 4.5 mmol/L c:  p= < 0.0001 d: p= <0.005 e: NS TC: Total Cholesterol; LDL-C: Low density lipoprotein; HDL: High-density lipoprotein; TG: Triglycerides; NS: Not Significant

Variable (mmol/L)	Baseline		6 months		12 months		Absolute reduction (6 months)		Absolute reduction (12 months)		% Reduction (6 months)		% Reduction (12 months)
Statin Tolerant (6months n= 57, 12 months n= 49)													
TC (mmol/L)	7.3 (6.7 – 7.9)	4.5 (4.1 – 4.9)		4.2 (3.7 – 4.8)		2.8 (2.3 – 3.2)		2.7 (2.1 – 3.3)		37.0 (32.3 – 41.7)^ c^		37.9 ( 30.7 – 45.1)^ c^	
LDL-C (mmol/L) (n=46^a^, n=40^b^)	4.9 (4.3 – 5.5)	2.2 (1.8 – 2.6)		2.1 (1.7 – 2.5)		2.7 (2.3 – 3.1)		2.6 (2.1 – 3.1)		55.2 (49.4 – 61.0)^ c^		53.6 (45.4 – 61.9)^ c^	
HDL-C (mmol/L)	1.3 (1.1 – 1.4)	1.3 (1.2 – 1.4)		1.3 (1.1 – 1.4)		-		-		-		-	
nHDL-C (mmol/L)	6.0 (5.4 – 6.7)	3.3 (2.8 – 3.7)		3.0 (2.4 – 3.5)		2.8 (2.3 – 3.1)		2.7 (2.2 – 3.3)		45.6 (39.7 – 51.6)^ c^		46.6 (38.1 – 55.0)^ c^	
TG (mmol/L)	2.1 (1.3 – 3.3)	1.6 (1.0 – 2.8)		1.4 (1.0 – 2.1)		0.2 (-0.1 – 0.9)		0.3 (-0.1 – 1.3)		14.8 (-8.7 – 34.8)^ d^		21.7 (-11.1 – 46.1)^ d^	
Statin Intolerant (6months n= 98, 12 months = 77)													
TC (mmol/L)	7.4 (7.1 – 7.7)	4.9 (4.6 – 5.2)		4.9 (4.6 – 5.2)		2.5 (2.2 – 2.8)		2.4 (2.1 – 2.8)		33.1 (29.9 – 36.3)^ c^		31.7 (28.0 – 35.4)^ c^	
LDL-C (mmol/L) (n=90^a^, n=69^b^)	4.8 (4.5 – 5.2)	2.5 (2.2 – 2.8)		2.6 (2.2 – 2.9)		2.3 (2.0 – 2.6)		2.3 (2.0 – 2.6)		47.5 (42.9 – 52.3)^ c^		46.1 (40.7 – 51.5)^ c^	
HDL-C (mmol/L)	1.5 (1.4 – 1.6)	1.5 (1.4 – 1.6)		1.4 (1.3 – 1.5)		-		-		-		-	
nHDL-C (mmol/L)	5.9 (5.6 – 6.3)	3.4 (3.2 – 3.7)		3.5 (3.2 – 3.8)		2.5 (2.2 – 2.8)		2.4 (2.1 – 2.8)		41.2 (37.3 – 45.2)^ c^		39.1 (34.7 – 43.6)^ c^	
TG (mmol/L)	2.0 (1.4 – 3.4)	1.8 (1.3 – 2.9)		1.9 (1.3 – 2.8)		0.2 (-0.3 – 0.7)		0.2 (-0.4 – 0.8)		12.3 (-15.4 – 35.0)^ d^		14.4 (-28.5 – 32.4)^ e^	

**Table 7:** Efficacy outcomes on statin tolerant cohort based on concomitant use of Ezetimibe. a: 6-month cohort after excluding subjects with TG > 4.5 mmol/L b: 12-month cohort after excluding subjects with TG > 4.5 mmol/L c:  p= < 0.0001 d: p= <0.005 e: NS TC: Total Cholesterol; LDL-C: Low-density lipoprotein- cholesterol; HDL-C: High-density lipoprotein-cholesterol; TG: Triglycerides; NS: Not Significant

Variable (mmol/L)	Baseline	6 months	12 months	Absolute reduction (6 months)	Absolute reduction (12 months)	% Reduction (6 months)	% Reduction (12 months)
PCSK9i + Statins + Ezetimibe (6 months n= 31, 12 months n= 30)							
TC (mmol/L)	7.0 (6.0 – 7.9)	4.3 (3.7 – 5.0)	4.4 (3.6 – 5.3)	2.6 (2.1 – 3.1)	2.4 (1.7 – 3.1)	37.8 (31.1 – 42.5)^ c^	35.4 (26.2 – 44.6)^ c^
LDL-C (mmol/L) (n=26^a^, n= 26^b^)	4.2 (3.4 – 5.0)	2.0 (1.4 – 2.6)	2.3 (1.7 – 2.9)	2.5 (1.9 – 3.1)	2.3 (1.7 – 2.9)	57.9 (50.5 – 65.2)^ c^	49.7 (39.0 – 60.3)^ c^
HDL-C (mmol/L)	1.3 (1.1 – 1.4)	1.3 (1.1 – 1.4)	1.3 (1.1 – 1.4)	-	-	-	-
nHDL-C (mmol/L)	5.7 (4.7 – 6.6)	3.1 (2.4 – 3.7)	3.2 (2.3 – 4.0)	2.6 (2.2 – 3.1)	2.4 (1.7 – 3.1)	47.7 (41.5 – 53.0)^ c^	43.7 (33.2 – 54.1)^ c^
TG (mmol/L)	1.5 (1.0 – 3.0)	1.4 (1.0 – 2.8)	1.4 (1.1 – 2.0)	0.2 (-0.1 – 0.6)	0.3 (-0.2 – 0.9)	11.11 (-5.0 – 34.8)^ d^	20.0 (-17.1 – 33.3)^ d^
PCSK9i + Statin – Ezetimibe (6 months n=26, 12 months n=19)							
TC (mmol/L)	7.7 (6.9 – 8.5)	4.8 (4.2 – 5.3)	4.0 (3.4 – 4.6)	2.9 (2.1 – 3.8)	3.2 (2.2 – 4.2)	35.5 (25.4 – 45.6)^ c^	41.9 (29.5 – 54.3)^ c^
LDL-C (mmol/L) (n=20^a^, n = 14^b^)	5.4 (4.7 – 6.1)	2.5 (1.8 – 3.1)	1.8 (1.3 – 2.4)	2.9 (2.1 – 3.6)	3.1 (2.2 – 4.0)	52.6 (38.7 – 66.1)^ c^	61.0 (47.4 – 74.6)^ c^
HDL-C (mmol/L)	1.2 (1.1 – 1.4)	1.2 (1.1 – 1.4)	1.3 (1.1 – 1.5)	-	-	-	-
nHDL-C (mmol/L)	6.5 (5.7 – 7.2)	3.5 (2.9 – 4.1)	2.7 (2.1 – 3.3)	2.9 (2.1 – 3.8)	3.3 (2.3 – 4.3)	43.2 (32.0 – 54.5)^ c^	51.1 (35.7 – 66.5)^ c^
TG (mmol/L)	2.2 (1.4 – 4)	1.7 (1.1 – 2.7)	1.4 (1.0 – 2.6)	0.4 (-0.2 – 2)	0.7 (0.0 – 2.6)	19.9 (-12.3 – 33.3)^ e^	30.2 (-1.4 – 56.8)^ d^

**Table 8:** Efficacy outcome in statin intolerance with or without Ezetimibe a: 6-month cohort after excluding subjects with TG > 4.5 mmol/L b: 12-month cohort after excluding subjects with TG > 4.5 mmol/L c:  p= < 0.0001 d: p= <0.005 e: NS f: p= <0.05 TC: Total Cholesterol; LDL-C: Low-density lipoprotein- cholesterol; HDL-C: High-density lipoprotein-cholesterol; TG: Triglycerides; NS: Not Significant

Variable (mmol/L)	Baseline	6 months	12 months	Absolute reduction (6 months)	Absolute reduction (12 months)	% Reduction (6 months)	% Reduction (12 months)
PCSK9i + Ezetimibe - Statins (6 months n= 28, 12 months n=23)							
TC (mmol/L)	7.2 (6.5 – 8.0)	5.0 (4.4 – 5.6)	4.5 (3.9 – 5.0)	2.3 (1.7 – 2.8)	2.6 (1.9 – 3.4)	30.3 (23.6 – 36.9)^ c^	34.3 (25.9 -42.6)^ c^
LDL-C (mmol/L) (n=26^a^, n=20 ^b^)	4.6 (3.9 – 5.3)	2.6 (2.0 – 3.2)	2.3 (1.8 -2.9)	2.0 (1.5 – 2.5)	2.2 (1.5 – 2.9)	42.3 (31.8 – 52.8)^ c^	45.3 (33.2 – 57.3)^ c^
HDL-C (mmol/L)	1.5 (1.3 – 1.7)	1.5 (1.3 – 1.7)	1.3 (1.2 -1.5)	-	-	-	-
nHDL-C (mmol/L)	5.7 (5.0 – 6.5)	3.4 (2.8 – 4.0)	3.1 (2.6 – 3.7)	2.3 (1.7 – 2.9)	2.6 (1.8 – 3.4)	39.1 (30.8 – 47.3)^ c^	42.1 (32.2 – 50.0)^ c^
TG (mmol/L)	1.9 (1.1 – 2.8)	1.3 (1.0 – 1.9)	1.3 (1.1 -2.7)	0.5 (0.1 – 0.8)	0.5 (0.0 – 0.9)	33.3 (2.9 – 43.2)^ d^	23.8 (0.0 -31.6)^ f^
PCSK9i – Ezetimibe - Statins (6 months n=70, 12 months n=54)							
TC (mmol/L)	7.5 (7.1 – 7.8)	4.9 (4.5 – 5.2)	5.1 (4.7 – 5.4)	2.6 (2.3 – 2.9)	2.4 (2.0 – 2.7)	34.2 (30.6 – 38)^ c^	30.6 (26.5 – 34.7)^ c^
LDL-C (mmol/L) (n=64^a^, n =49^b^)	4.9 (4.5 – 5.3)	2.5 (2.2 – 2.8)	2.6 (2.2 -3.0)	2.4 (2.1 – 2.7)	2.3 (2.0 – 2.7)	49.7 (44.6 – 54.8)^ c^	46.5 (40.3 – 52.6)^ c^
HDL-C (mmol/L)	1.4 (1.3 – 1.5)	1.4 (1.3 – 1.5)	1.4 (1.3 – 1.5)	-	-	-	-
n-HDL-C (mmol/L)	6.0 (5.7 – 6.4)	3.4 (3.1 – 3.7)	3.7 (3.3 – 4.0)	2.6 (2.3 – 2.9)	2.4 (2.0 – 2.7)	42.1 (37.5 – 46.7)^ c^	37.9 (33.0 – 42.8)^ c^
TG (mmol/L)	2.2 (1.5 – 3.6)	2.2 (1.4 – 3.0)	2.1 (1.7 – 3.1)	0.1 (-0.6 – 0.8)	0.2 (-0.6 – 0.8)	6.4 (-26.8 – 30.2)^ e^	7.7 (-30.4 -33.2)^ e^
